# Tonsillar Metastasis of Hepatocellular Carcinoma: A Case Report and Review of the Literature

**DOI:** 10.7759/cureus.58250

**Published:** 2024-04-14

**Authors:** Chalothorn Wannaphut, Toshiaki Takahashi, Sharina Macapagal, Manasawee Tanariyakul, Sorawit Ongsupankul, Thanaboon Yinadsawaphan, Yoshito Nishimura, Jared Acoba

**Affiliations:** 1 Internal Medicine, University of Hawaii John A. Burns School of Medicine, Honolulu, USA; 2 Internal Medicine, University of Hawaii, Honolulu, USA; 3 Hematology and Oncology, The Queen's Medical Center, Honolulu, USA

**Keywords:** oral bleeding, head and neck neoplasms, tonsillar metastasis, extrahepatic metastasis, hepatocellular carcinoma

## Abstract

A 63-year-old male with stage IV hepatocellular carcinoma (HCC), accompanied by lung and adrenal metastases, presented with oral bleeding. Physical examination disclosed bleeding from the tonsillar mass. A head and neck computed tomography identified a 2.4 cm enhancing lesion in the right anterior ethmoidal sinus, extending to the nasal region and medial orbit. Tonsillar mass biopsy confirmed HCC metastasis, immunopositive for Hepatocyte Paraffin 1 (HepPar1) and Arginase. He was treated with local radiotherapy (30 fractions). The unique presentation of severe bleeding from a tonsillar biopsy-proven HCC metastatic lesion underscores the rarity of head and neck involvement. Extrahepatic metastasis, particularly to the head and neck area likely due to hematogenous spread, may be a major independent predictor of poor outcomes in HCC patients. Local radiotherapy to achieve local hemostasis and reduce tumor bulk should be considered. In patients with known HCC having new oropharyngeal symptoms, HCC metastasis should be considered for a timely diagnosis. Despite its rarity, this manifestation signifies an unfavorable prognosis, reinforcing the imperative for a multidisciplinary approach to enhance therapeutic outcomes in these complex scenarios.

## Introduction

Hepatocellular carcinoma (HCC) ranks as the sixth most common cancer globally and stands as the third leading cause of cancer mortality with almost 830,200 deaths each year [1]. Prognostic factors include the severity of liver disease, tumor characteristics, and metastases. Extrahepatic metastasis for HCC occurs in about 13% of the cases. The most common sites of extrahepatic metastases are the lung, intra-abdominal lymph nodes, bone, and adrenal gland [2]. However, head and neck metastases are very uncommon. Here, we present the case of a patient with advanced-stage HCC with unusual metastases to the anterior ethmoid sinus, medial orbit, and palatine tonsil, leading to severe oropharyngeal bleeding as an initial presentation.

## Case presentation

The patient is a 63-year-old male with stage Barcelona Clinical Liver Cancer (BCLC) stage C HCC with known lung and adrenal metastases with prior liver resection and left adrenalectomy, cirrhosis due to hepatitis C infection, and pulmonary embolism on rivaroxaban. The patient presented to the emergency department with painless oral bleeding. He reported no fever, cough, dyspnea, or dysphagia. While the initial differential diagnosis included hemoptysis and gastrointestinal bleeding, a physical examination revealed that the source of bleeding was the tonsillar mass.

**Figure 1:**
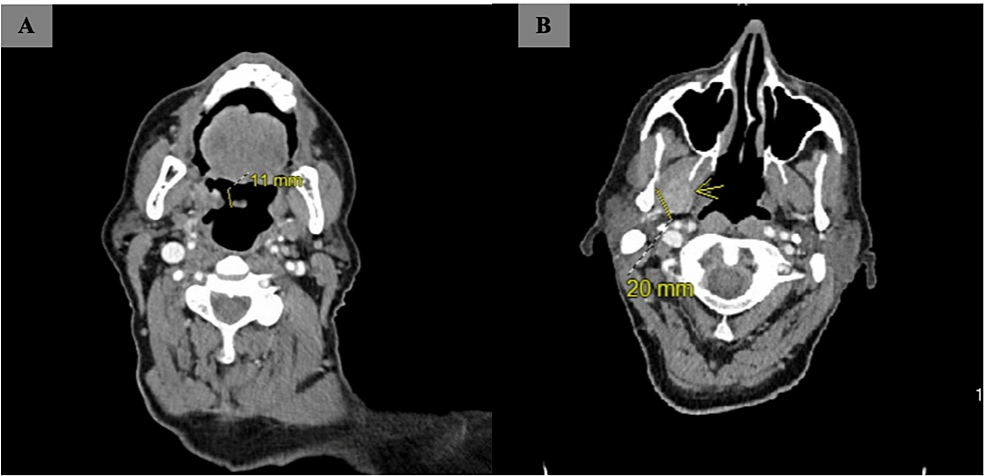
Pre-treatment computed tomography of the head and neck with contrast Pre-treatment computed tomography of the head and neck with contrast; (A) Exophytic nodule 1.1 cm involved the right palatine tonsil, (B) Enhancing lesion 2 cm in the right masticator space affected the medial and lateral pterygoid muscles

**Figure 2:**
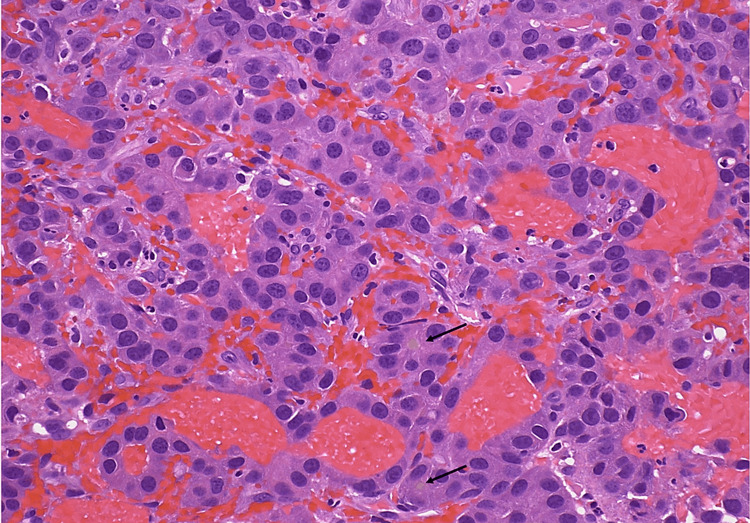
Histopathology photographs of tonsil metastatic lesions (magnification 200×) with trabecular architecture with sinusoid formation and focal bile pigment (arrows).

**Figure 3:**
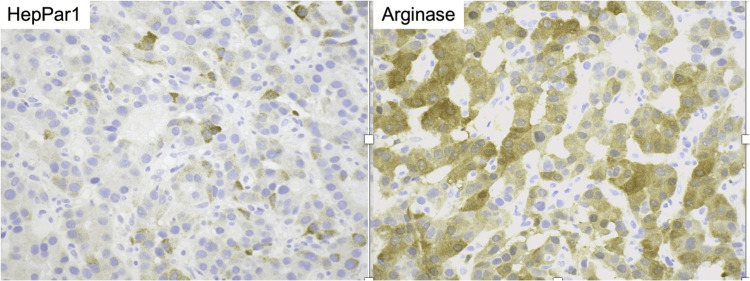
Immunohistochemistry positive for HepPar 1 (tonsil metastatic lesion) (magnification 200×). Immunohistochemistry positive for arginase (tonsil metastatic lesion) (magnification 200×). HepPar1: Hepatocyte Paraffin 1

The results of the main laboratory testing were summarized in Table 1 with initial findings indicating hemoglobin levels of 7.3 g/dl (baseline 10.0 g/dL two months before the presentation), platelet count at 365,000 cell/mm3, partial thromboplastin time at 39.8 secs, prothrombin time at 23.6 secs, and international normalised ratio (INR) of 2.2. Liver function tests revealed aspartate aminotransferase 50 IU/L, alanine transaminase 36 IU/L, alkaline phosphatase 182 IU/L, albumin 3.4 g/dl, total bilirubin 0.8 mg/dl, and pretreatment alpha-fetoprotein of 3074 ng/ml. Head, neck, and chest computed tomography (CT) identified a 2.4 cm enhancing lesion in the right anterior ethmoidal sinus, extending to the nasal region and medial orbit. Additionally, a 1.1 cm exophytic nodule involved the right palatine tonsil (Figure 1A), and a 2.0 cm enhancing lesion in the right masticator space affected the medial and lateral pterygoid muscles (Figure 1B) without evidence of pulmonary embolism. Homeostasis was successfully achieved by transamine nebulization and topical nitrate application. A tonsillar mass biopsy showed trabecular architecture with the sinusoid formation and focal bile pigment (Figure 2) with immunopositive for Hepatocyte Paraffin 1 (HepPar1) and Arginase (Figure 3), but negative for Glypican-E, p40, and Cytokeratins 7 and 20, which confirmed HCC metastases.

**Table 1:** Main laboratory data Abbreviations: Hb: Hemoglobin, WBC: White Blood Cell, Hct: Hematocrit Plt: Platelet, Na: Sodium, K: Potassium, Cl; Chloride, S.Cr: Serum creatinine, TP: Total protein, Alb: Albumin, AST: Aspartate Aminotransferase, ALT: Alanine Aminotransferase, ALP: Alkaline Phosphatase, T-Bil: Total Bilirubin, PT: Protime, PTT: Partial Thromboplastin time, INR: International normalized ratio

Complete blood count	Result	Reference range
WBC (10^3 ^/uL)	9.8	3.8-10.84
Hb (g/dl)	7.3	11.2-15.7
Hct (%)	26.1	34.1-44.9
Plt (10^3 ^/uL)	365	151-424
Biochemical findings		
Na (mEq/L)	133	133-145
K (mEq/L)	3.5	3.3-5.1
Cl (mEq/L)	88	95-108
Glucose (mg/dL)	97	70-99
BUN (gm/dL)	15	6.0-23
S.Cr (gm/dL)	1.2	0.6-1.4
TP (gm/dL)	7.6	6.4-8.3
Alb (mg/dL)	3.4	3.5-5.2
AST (IU/L)	50	<40
ALT (IU/L)	36	<42
ALP (IU/L)	182	35-129
T.Bil (mg/dL)	0.8	<1.2
Coagulation		
PT (secs)	23.6	11.8-14.2
PTT	39.8	23.5-37.8
INR	2.2	<1.1
Pretreatment Alpha fetoprotein (ng/ml)	3074	5.0-10.0
Posttreatment Alpha fetoprotein (ng/ml)	17	5.0-10.0

**Figure 4:**
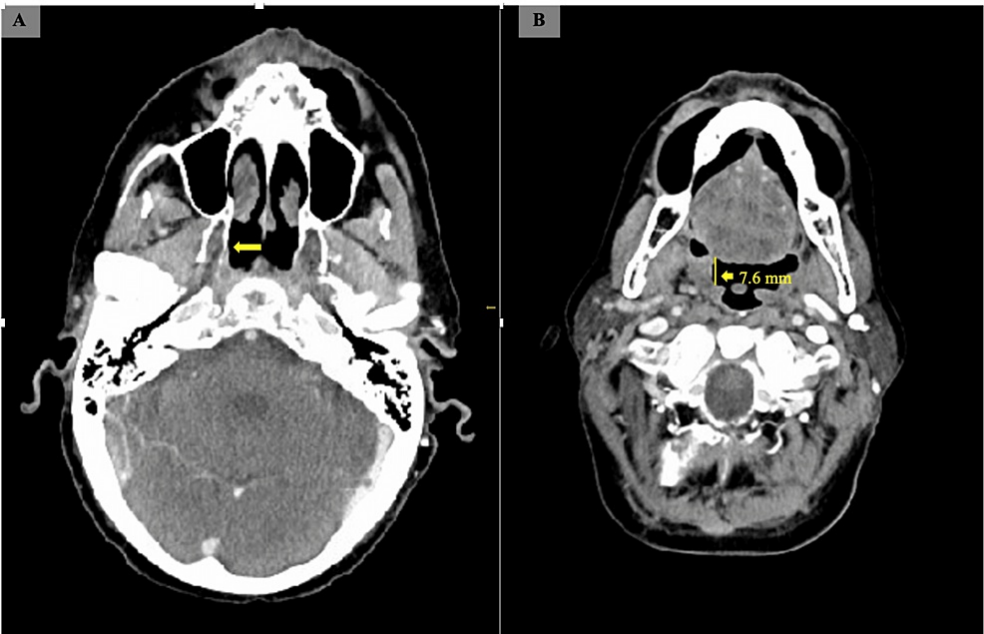
Post-treatment computed tomography of the head and neck with contrast Post-treatment computed tomography of the head and neck with contrast (palatine tonsil): (A) Resolution of lesions at right masticator space and pterygoid muscle, (B) Decreasing nodule size of about 50% at the right palatine tonsil.

The patient was initiated on a course of stereotactic body radiation therapy comprising 30 fractions, targeting metastases in the right ethmoid, frontal, tonsillar, and masticator space. He had previously received nivolumab with the resolution of his lung metastases but complicated by immune-mediated pneumonitis. He tolerated the radiation well without significant acute side effects. Surveillance of alpha-fetoprotein (AFP) levels has demonstrated a consistent and favorable downward trend from 3074 to 17 ng/ml. After radiation, the patient was started on cabozantinib. After three months of treatment, repeat imaging showed the resolution of lesions at the right masticator space and pterygoid muscle (Figure 4A) and a decreasing nodule size of about 50% at the right palatine tonsil (Figure 4B).

## Discussion

We reported a rare case of HCC with metastasis to a tonsil leading to severe oropharyngeal bleeding. To date, four cases of tonsillar metastasis of HCC have been reported in the English literature (Table 2)[3-6]. In all patients, sites of metastasis were palatine tonsils, and other metastases included the rib, gingiva, and lung. Two cases presented with tonsillar mass, one with tonsillar bleeding, and the other with dysphagia. While the mechanism of distant metastasis is unclear, it is speculated that it is most likely from a hematogenous route, as suggested by a previous report that tumor cells could reach the head and neck lesion by bypassing the lung via the venous plexus of Batson [7].

**Table 2:** Prior case reports of four patients with hepatocellular carcinoma metastasis to the tonsils HBV: Hepatitis B virus; HCV: Hepatitis C virus

First author/ year	Age	Gender	Underlying condition	Symptoms/signs	Other metastasis	Treatment for metastasis	Survival after treatment
Lianes et al., 1996 [3]	71	Male	HBV&HCV	Lump	None	Tonsillectomy	10 months
Endo et al., 2006 [4]	58	Male	NA	Bleeding	Lung	Tonsillectomy	NA
Wen et al., 2008 [5]	66	Male	NA	Lump	None	Tonsillectomy	9 months
Karamitsou et al., 2023 [6]	84	Male	HBV	Dysphagia	Rib	None	6 months

According to the latest National Comprehensive Cancer Network guidelines, systemic therapy with the combination of atezolizumab plus bevacizumab (for Child-Pugh Class A HCC with satisfactory performance status), and the combination of tremelimumab-actl plus durvalumab are the first-line treatment for patients with unresectable disease, comorbidity, or metastatic disease. When patients are deemed with poor tolerance to dual immunotherapy, monotherapy with sorafenib or lenvatinib is preferred. The subsequent systemic therapy options for disease progression include multikinase inhibitors such as regorafenib, cabozantinib, lenvatinib, and sorafenib [8]. If the first-line therapy was sorafenib or lenvatinib, the second-line options were regorafinib, cabozantinib, or ramucirumab (if AFP >400 ng/ml). In our case, the patient was initially treated with radiation to address the bleeding and provide symptomatic relief. His initial presentation of metastatic disease was before the availability of combination therapy with either atezolizumab and bevacizumab, or tremilimumab and durvalumab, and he was treated with sorafenib followed by nivolumab. Given his prior immunotherapy and immune-mediated pneumonitis, he was started on cabozantinib following his radiation.

The prognosis of patients with tonsillar metastases from HCC is not well defined due to the limited number reported to date. According to the study of Schutte et al., patients with HCC with distant metastases had a median survival of 11.3 months, while those without metastasis had a median survival of 14.8 months [9]. Our literature review also revealed that the cases with tonsillar metastasis demonstrated a poor outcome with a survival duration of less than 12 months. Overall, given the potentially poor overall survival of the patients with tonsilar metastases from HCC, it is essential to control complications such as impaired quality of life, malnutrition due to dysphagia, as well as bleeding from the metastatic lesion, as in our case. Stereotactic body radiation therapy (SBRT) is used for patients with one to three primary sites and larger hepatic lesions, and is also used as palliative radiation therapy for metastatic HCC lesions involving the bone or brain [10]. In addition to the targeted molecular therapy and immunotherapy described above, radiotherapy can also be a critical element in formulating a treatment strategy for HCC patients with oral metastasis.

## Conclusions

In patients with known HCC experiencing new oropharyngeal symptoms, the possibility of HCC metastasis should be promptly considered for timely diagnosis. Despite its rarity, this manifestation indicates an unfavorable prognosis, emphasizing the necessity of a multidisciplinary approach to optimize therapeutic outcomes in such complex scenarios. Our case highlights the importance of recognizing atypical presentations in advanced-stage HCC, such as metastases to the head and neck region. We achieved symptomatic relief and managed disease progression effectively by combining radiation therapy, targeted molecular therapy such as cabozantinib, and prior immunotherapy. While the prognosis for HCC patients with tonsillar metastases remains challenging, aggressive management strategies, including innovative treatments like stereotactic body radiation therapy, are crucial for controlling complications and extending survival. Further research and clinical experience are vital to better understand and improve outcomes for patients with similar presentations of HCC metastasis.
